# Supplementary material for: MetaRibo-Seq measures translation in microbiomes
Source: Nat Commun. 2020 Jun 29;11:3268. doi: 10.1038/s41467-020-17081-z (PMC7324362; doi:10.1038/s41467-020-17081-z)
Supplement: Supplementary file 10 — Supplementary Data 7 [file 41467_2020_17081_MOESM10_ESM.zip › File2/Confidence_VeryHigh_Taxonomy/200058_out.krona.html]

Javascript must be enabled to view this page.

members
magnitude
magnitudeUnassigned
count
unassigned
taxon
rank

200058\_out

24

2
superkingdom
24

1239
phylum
23

23
class
186801


SRS053356\_contig\_number\_contig-100\_29058.115094
order
1
23
186802

21
species

SRS011134\_contig\_number\_28294SRS011302\_contig\_number\_13720SRS014235\_contig\_number\_15779SRS020394\_contig\_number\_15041SRS020869\_contig\_number\_contig-100\_10136.161981SRS022524\_contig\_number\_6587SRS024132\_contig\_number\_contig-100\_2757.298013SRS024435\_contig\_number\_40602SRS048870\_contig\_number\_11871SRS052697\_contig\_number\_19477SRS064757\_contig\_number\_20867SRS077849\_contig\_number\_contig-100\_22794.58984SRS078419\_contig\_number\_contig-100\_33258.114589SRS1041033\_contig\_number\_7526SRS1041137\_contig\_number\_contig-100\_2860.166531SRS104636\_contig\_number\_29967SRS144135\_contig\_number\_13426SRS146812\_contig\_number\_contig-100\_2848.269423SRS147139\_contig\_number\_36628SRS147557\_contig\_number\_31984SRS150029\_contig\_number\_20506
1898207

family
1
31979

1485
genus
1


SRS098571\_contig\_number\_contig-100\_21865.233850
species
1
1262841

544448
1
phylum

31969
class
1

2085
order
1

2092
1
family

genus
1
2093

1262904

SRS013476\_contig\_number\_37110
species
1
